# Supplementary material for: Structural and functional determination of peptide versus small molecule ligand binding at the apelin receptor
Source: Nat Commun. 2024 Dec 27;15:10714. doi: 10.1038/s41467-024-55381-w (PMC11680790; doi:10.1038/s41467-024-55381-w)
Supplement: Supplementary file 1 — Supplementary Information [file 41467_2024_55381_MOESM1_ESM.pdf]

Supplementary Information

Structural and functional determination of peptide versus small molecule ligand binding at the apelin receptor

Thomas L. Williams[1*], Grégory Verdon[2*], Rhoda E. Kuc[1], Heather Currinn[2], Brian Bender[2], Nicolae Solcan[2], Oliver Schlenker[2], Robyn G.C. Macrae[1,3], Jason Brown[2], Marco Schütz[2], Andrei Zhukov[2], Sanjay Sinha[3], Chris de Graaf[2§], Stefan Gräf[4,5,6§], Janet J. Maguire[1§], Alastair J.H. Brown[2§], Anthony P. Davenport[1§]

1. Experimental Medicine & Immunotherapeutics, University of Cambridge, Cambridge CB2 0QQ, UK
2. Nxera Pharma UK (Sosei Heptares), Steinmetz Building, Granta Park, Cambridge CB21 6GT, UK
3. Wellcome-MRC Cambridge Stem Cell Institute, Jeffrey Cheah Biomedical Centre, University of Cambridge, Cambridge CB2 0AW, UK
4. NIHR BioResource for Translational Research – Rare Diseases, Cambridge Biomedical Campus, Cambridge CB2 0QQ, UK
5. Department of Haematology, NHS Blood and Transplant, Long Road, University of Cambridge, Cambridge CB2 0PT, UK
6. Victor Phillip Dahdaleh Heart and Lung Research Institute, University of Cambridge, Cambridge Biomedical Campus, Cambridge CB2 0BB, UK

* These authors contributed equally

[§] These authors jointly supervised this work

Author(s) for Correspondence:

Anthony P. Davenport (apd10@medschl.cam.ac.uk)
Experimental Medicine and Immunotherapeutics
University of Cambridge
Level 6, Addenbrooke's Centre for Clinical Investigation (ACCI)
Box 110, Addenbrooke's Hospital, Cambridge, UK, CB2 0QQ

Alastair J.H. Brown (Alastair.Brown@ Nxera.life)
Nxera.Life (Sosei Heptares)
Steinmetz Building
Granta Park, Cambridge, UK, CB21 6GT

Supplementary Figures

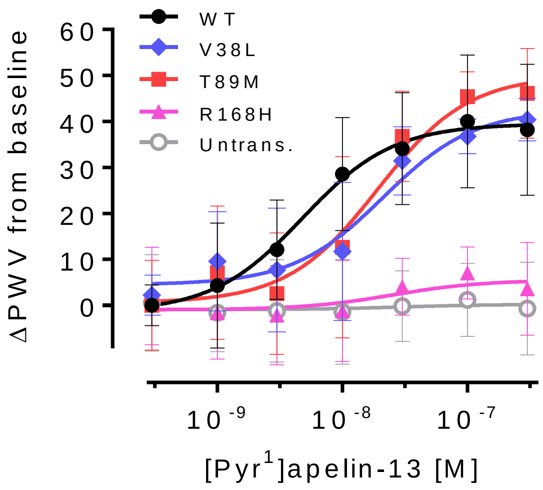

Fig. S1: The R/H168$^{4.64}$ apelin receptor variant is unresponsive to [Pyr^{1}]apelin-13 in a functional dynamic mass redistribution assay. Concentration-response curves showing response to endogenous [Pyr^{1}]apelin-13 peptide in CHO-K1 cells transfected with wild-type or variant apelin receptor (as indicated in figure). ΔPWV = change in peak wavelength value. WT = cells transfected with wild-type apelin receptor. Colours indicate the missense (V/L381.42, blue; T/M892.64, red; R/H1684.64, magenta). Untrans. = untransfected cells. Data are expressed as mean ± SD, n = 3 independent experiments. Source data are provided as a Source Data file.

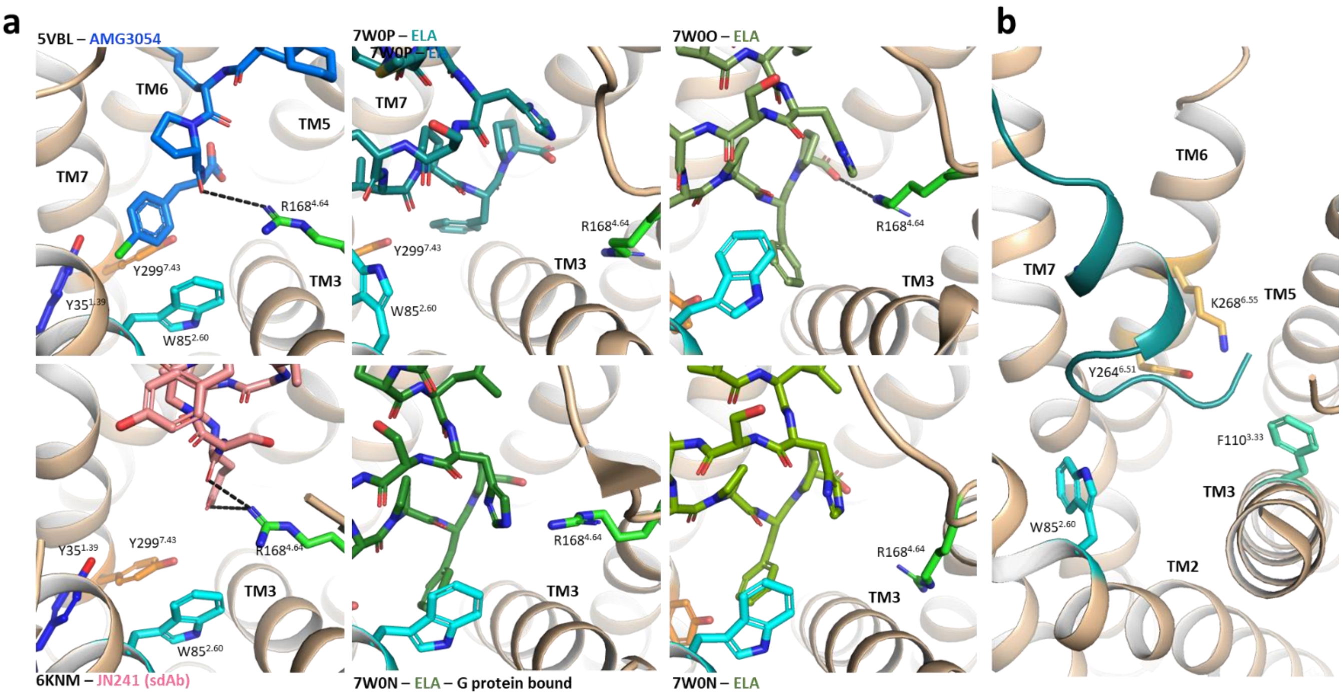

Fig. S2. Close-up view of peptide C-termini and relative position of R168$^{4.64}$ side chain in previously reported crystal and cryoEM structures of the human apelin receptor. a Cartoon representation of previously reported apelin receptor structures bound to bound the synthetic agonist peptide AMG3054 (top left), synthetic single-domain antibody (sdAb) antagonist JN241 (bottom left), and to ELA (middle and right panels); **b** Cartoon representation of ELA engagement to the apelin receptor in 7W0P.

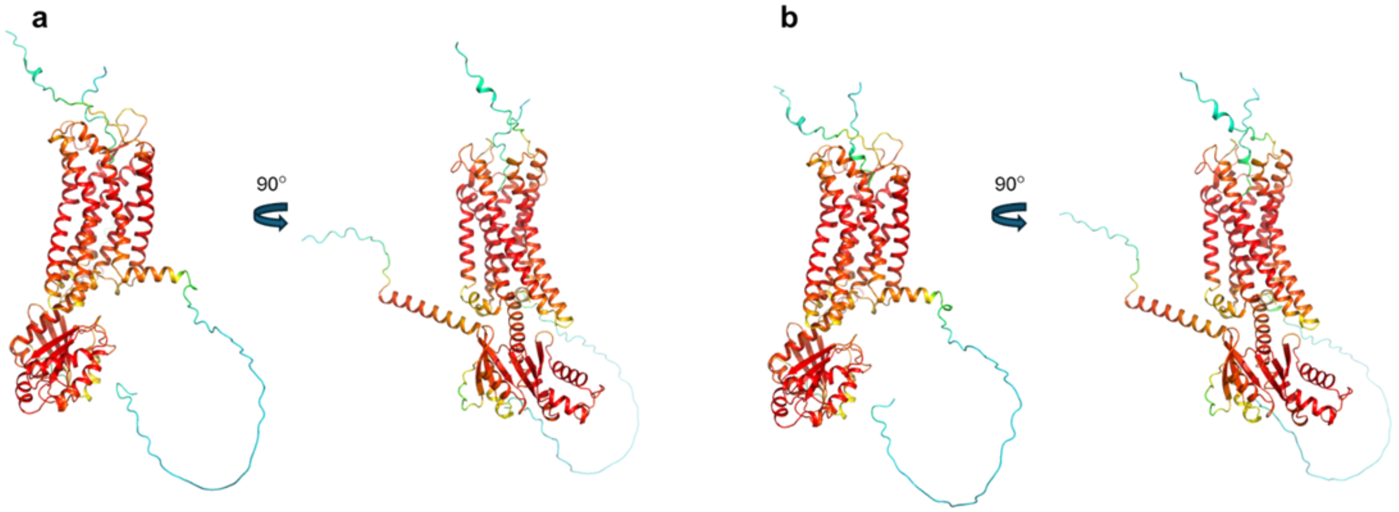

Fig. S3A: Cartoon representation of AF2 models of APJ and G protein alpha complex bound to apelin and (a, left) and Elabela (b, right) in two different orientations, and with the local confidence scores (pLDDT) plotted in rainbow colours from dark blue (Very low confidence, pLDDT < 50) to dark red (Very high confidence, pLDDT > 90).

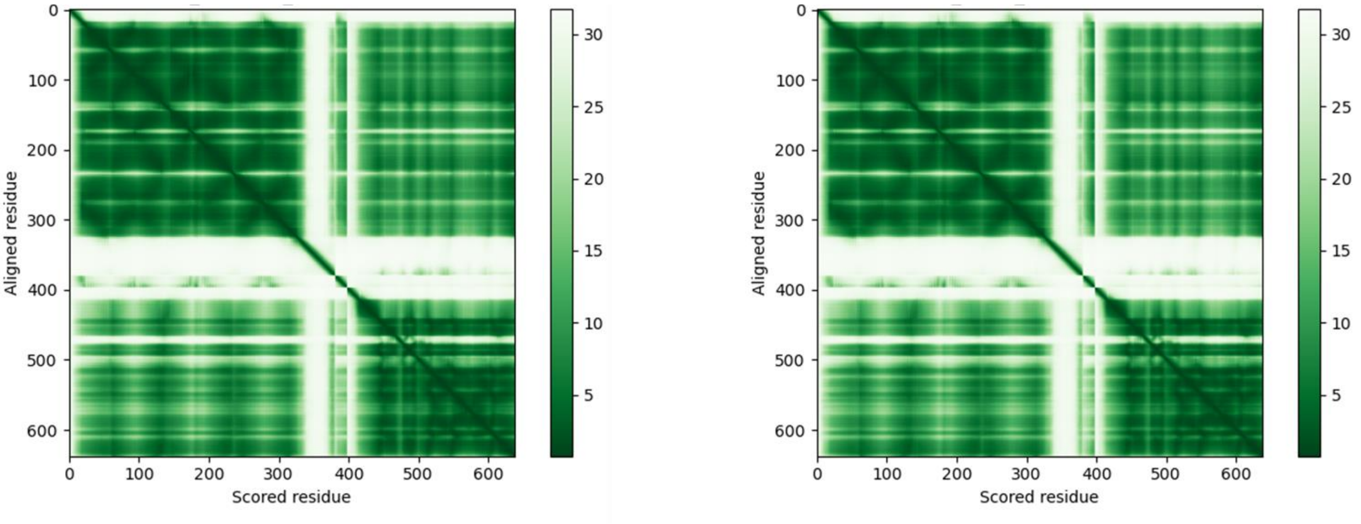

Fig. S3B: Predicted Aligned Error (PAE) plots for AF2 models of APJ and G protein alpha complex bound to apelin and (left) and Elabela (right) (17 residues each), with the scale indicating the expected position error (Ångströms).

Fig. S3C Sequences of Apelin, Elabela, apelin receptor and Guanine nucleotide-binding protein G(i)subunit alpha-1 (from Uniprot, https://www.uniprot.org/) used in the AlphaFold model.

Apelin (Q9ULZ1, APEL_HUMAN)SEQUENCE USED:

KFRRQRPRLSHKGPMPF

Elabela (P0DMC3, ELA_HUMAN)SEQUENCE USED:

NCLQRRCMPLHSRVPFP

Apelin receptor (P35414, APJ_HUMAN)SEQUENCE USED:

MEEGGDFDNYYGADNQSECEYTDWKSSGALIPAIYMLVFLLGTTGNGLVLWTVFRSSREKRRSADIFIAS

LAVADLTFVVTLPLWATYTYRDYDWPFGTFFCKLSSYLIFVNMYASVFCLTGLSFDRYLAIVRPVANARL

RLRVSGAVATAVLWVLAALLAMPVMVLRTTGDLENTTKVQCYMDYSMVATVSSEWAWEVGLGVSSTTVGF

VVPFTIMLTCYFFIAQTIAGHFRKERIEGLRKRRRLLSIIVVLVVTFALCWMPYHLVKTLYMLGSLLHWP

CDFDLFLMNIFPYCTCISYVNSCLNPFLYAFFDPRFRQACTSMLCCGQSRCAGTSHSSSGEKSASYSSGH

SQGPGPNMGKGGEQMHEKSIPYSQETLVVD

Apelin receptor (P35414, APJ_HUMAN) T/M89$^{2.64}$ SEQUENCE USED:

MEEGGDFDNYYGADNQSECEYTDWKSSGALIPAIYMLVFLLGTTGNGLVLWTVFRSSREKRRSADIFIAS

LAVADLTFVVTLPLWATY**M**YRDYDWPFGTFFCKLSSYLIFVNMYASVFCLTGLSFDRYLAIVRPVANARL

RLRVSGAVATAVLWVLAALLAMPVMVLRTTGDLENTTKVQCYMDYSMVATVSSEWAWEVGLGVSSTTVGF

VVPFTIMLTCYFFIAQTIAGHFRKERIEGLRKRRRLLSIIVVLVVTFALCWMPYHLVKTLYMLGSLLHWP

CDFDLFLMNIFPYCTCISYVNSCLNPFLYAFFDPRFRQACTSMLCCGQSRCAGTSHSSSGEKSASYSSGH

SQGPGPNMGKGGEQMHEKSIPYSQETLVVD

Guanine nucleotide-binding protein G(i) subunit alpha-1 (P63096, GNAI1_HUMAN)SEQUENCE USED:

MGHHHHHHENLYFQGTLSAEDKAAVERSKMIDRNLREDGEKAAREVKLLLLGADNSGKSTIVKQMKIIHG

GGGGGGGTTGIVETHFTFKDLHFKMFDVGGQRSERKKWIHCFEDVAAIIFCVDLSDYNRMHESMKLFDSI

CNNKWFTDTSIILFLNKKDLFEEKIKKSPLTICYQEYAGSNTYEEAAAYIQCQFEDLNKRKDTKEIYTHF

TCATDTKNAQFIFDAVTDVIIKNNLKDCGLF

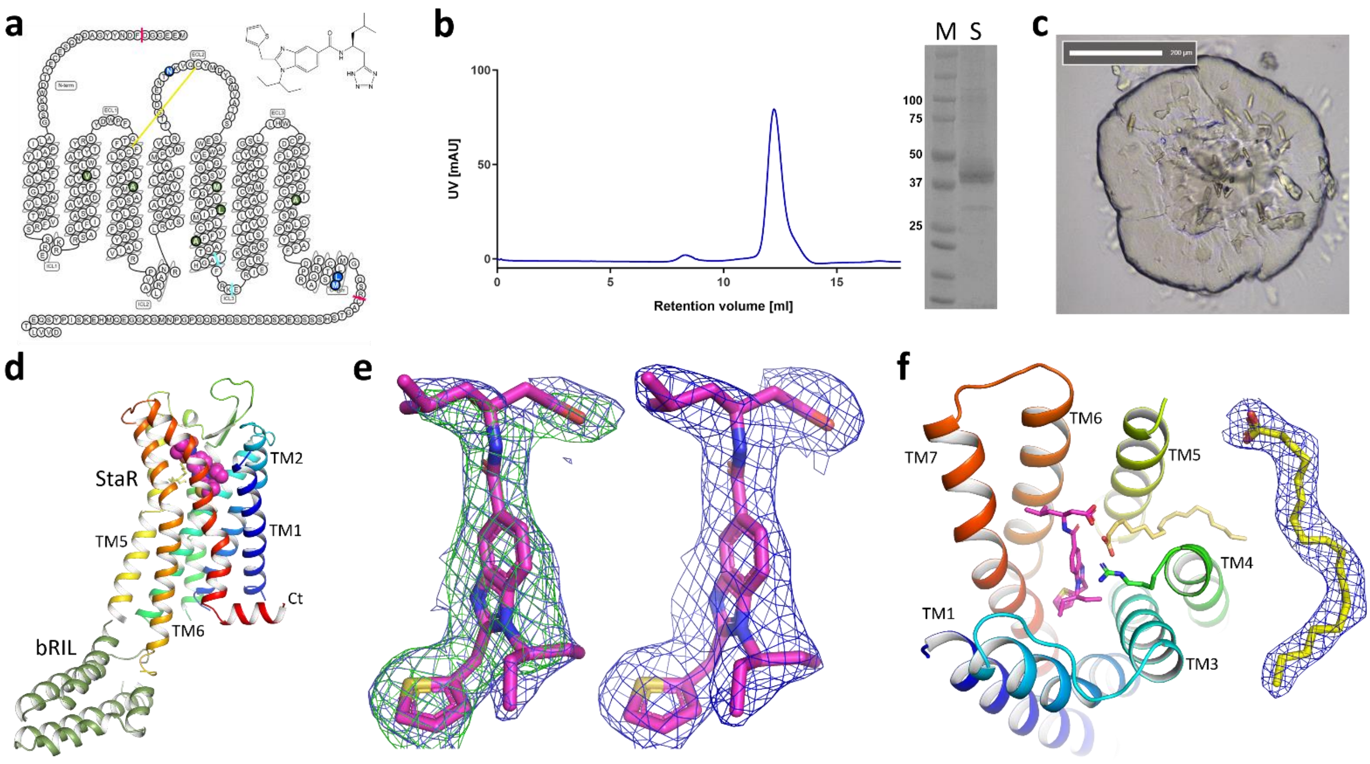

Fig. S4: Apelin receptor NxStaR generation to crystal structure of with the small synthetic G protein-biased agonist CMF-019. a Snake plot of the protein sequence of the apelin receptor NxStaR used for the crystal structure determination as generated by GPCRdb (https//:gpcrdb.org). The 6 thermostabilising and 3 post-translational modification removal mutations are highlighted in green and blue, respectively. The two cysteine residues making a disulfide bond are linked with a solid yellow line. N- and C-terminal truncation and optimised bRIL fusion insertion points are highlighted with small red and cyan bars, respectively. **b** UV absorption profile at 280 nm of the elution crystallisation construct from a SEC200 Sephadex 10/300 column (left), and SDS-PAGE analysis of a sample used for crystallisation (S) along molecular weight markers (M), for which relevant values in kDa on the left (right). **c** Bright-field image of a bolus of mesophase with crystals of the apelin receptor NxStaR bRIL CMF-019 complex. **d** Cartoon representation of the crystal structure of the apelin receptor NxStaR (rainbow) with the bRIL fusion (green) in complex with CMF-019 (spheres). **e** Residual 2*F*o-*F*c (blue mesh, 1σ) and *F*o-*F*c (green mesh, 3σ) electron density maps obtained after refinement of the apelin receptor NxStaR bRIL chimera without (left) and with CMF-019 (right) on the refined pose of CMF-019 in stick representation. **f** Top view of the apelin receptor NxStaR structure in cartoon representation with CMF-019 (magenta) and the molecule of oleic acid (yellow) represented as sticks, and 2*F*o-*F*c (blue mesh, 1σ) electron density map around the oleic acid molecule.

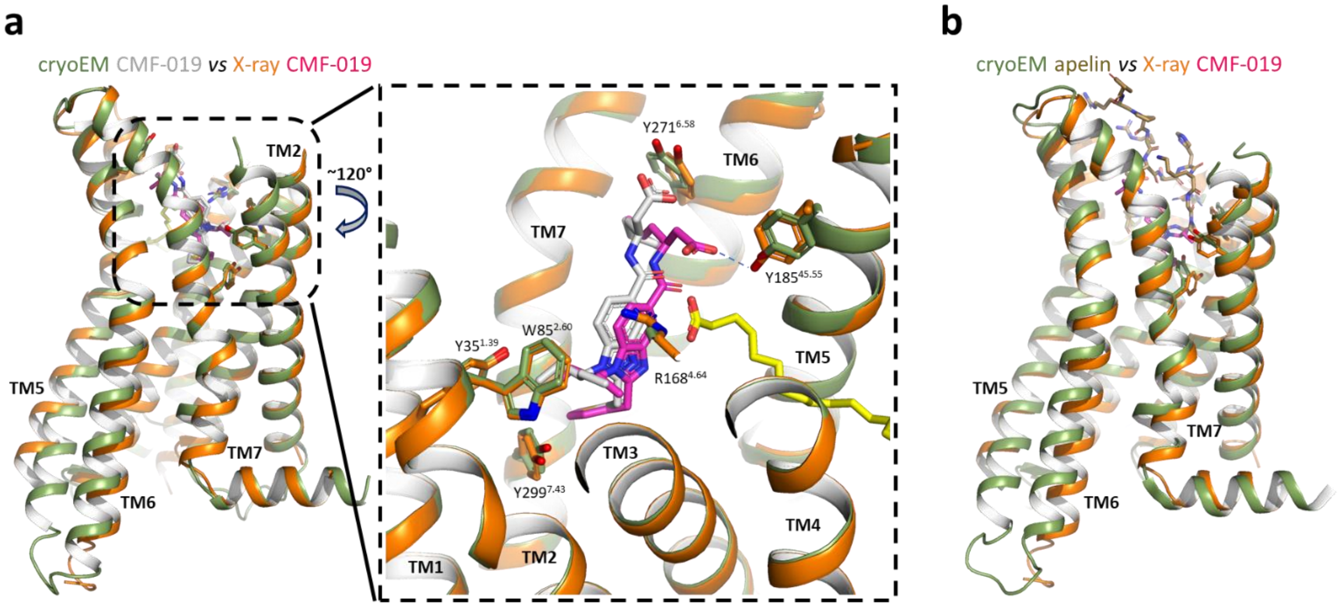

Fig. S5: Comparison of the apelin receptor CMF-019 crystal structure, versus apelin receptor CMF-019 and apelin cryoEM structures highlights the CMF-019 complex crystal structure's active-like conformation. Views of an overlay of apelin receptor NxStaR crystal structure (orange) in complex with CMF-019 (magenta) with **a** CMF-019 (grey) and **b** apelin (brown) cryoEM structures (green) in cartoon representation. Small molecule agonists and relevant amino acid side chains are represented as sticks.

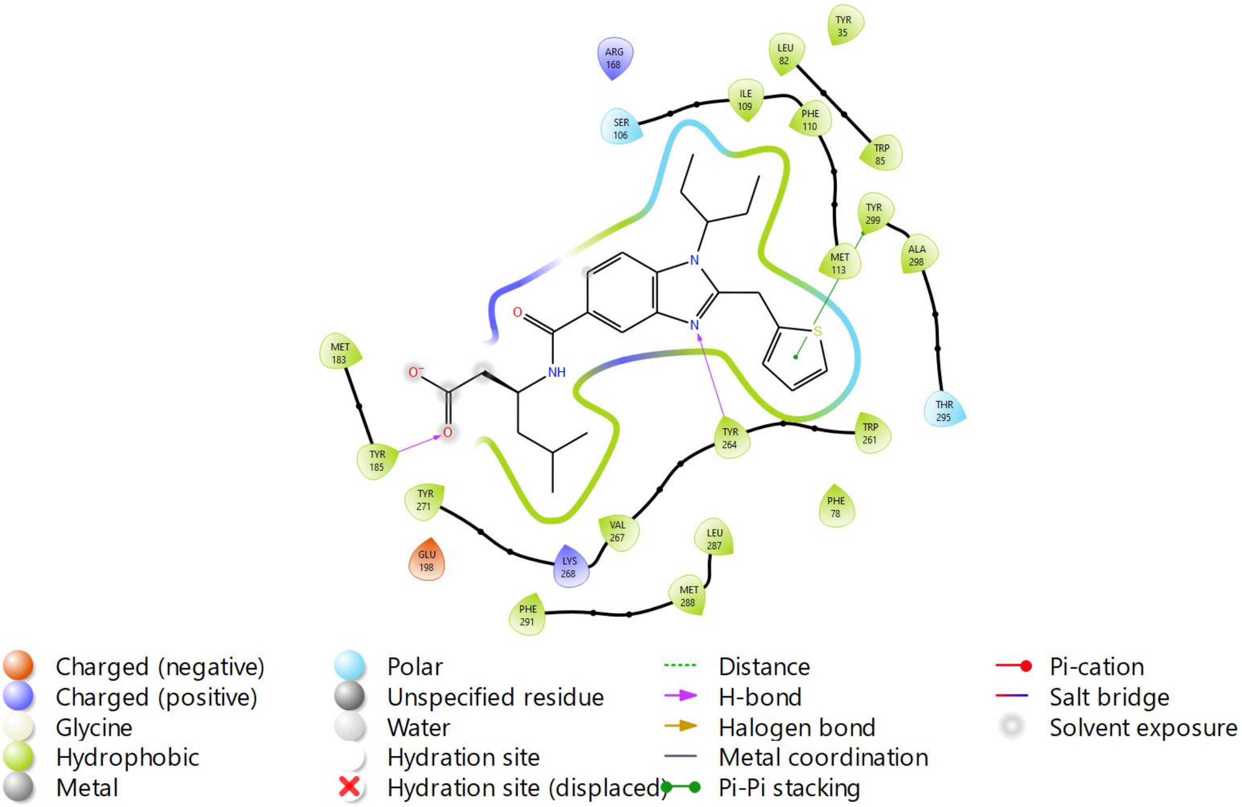

Fig. S6: of the amino acid and small synthetic, G protein-biased CMF-019 interactions in the apelin receptor NxStaR crystal structure.

Two-dimensional representation showing the principal interactions between the residues of CMF-019 and amino acids in the apelin receptor.

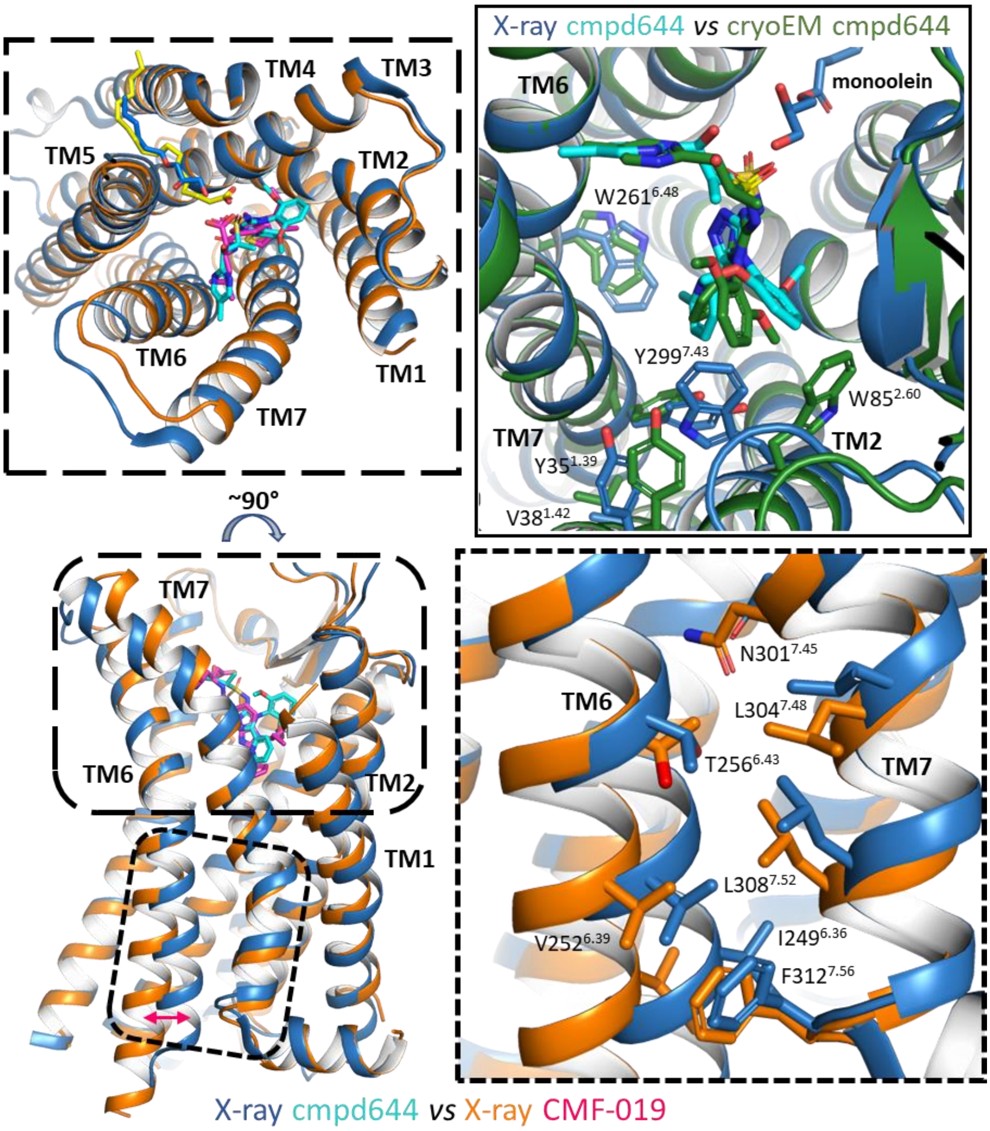

Fig. S7: Comparison of apelin receptor small synthetic agonist crystal structures highlights the CMF-019 complex structure's more active-like conformation. Views of an overlay of apelin receptor NxStaR crystal structure (orange) in complex with CMF-019 (magenta), with the cmpd644 crystal structure (cyan or green) in cartoon representation. Small molecule agonists and relevant amino acid side chains are represented as sticks. Left panels show top-down and side views, with close-up view (right panels) of TM6-TM7 interface, and cmpd644 binding poses (top-right) in previous crystal and cryoEM structures.

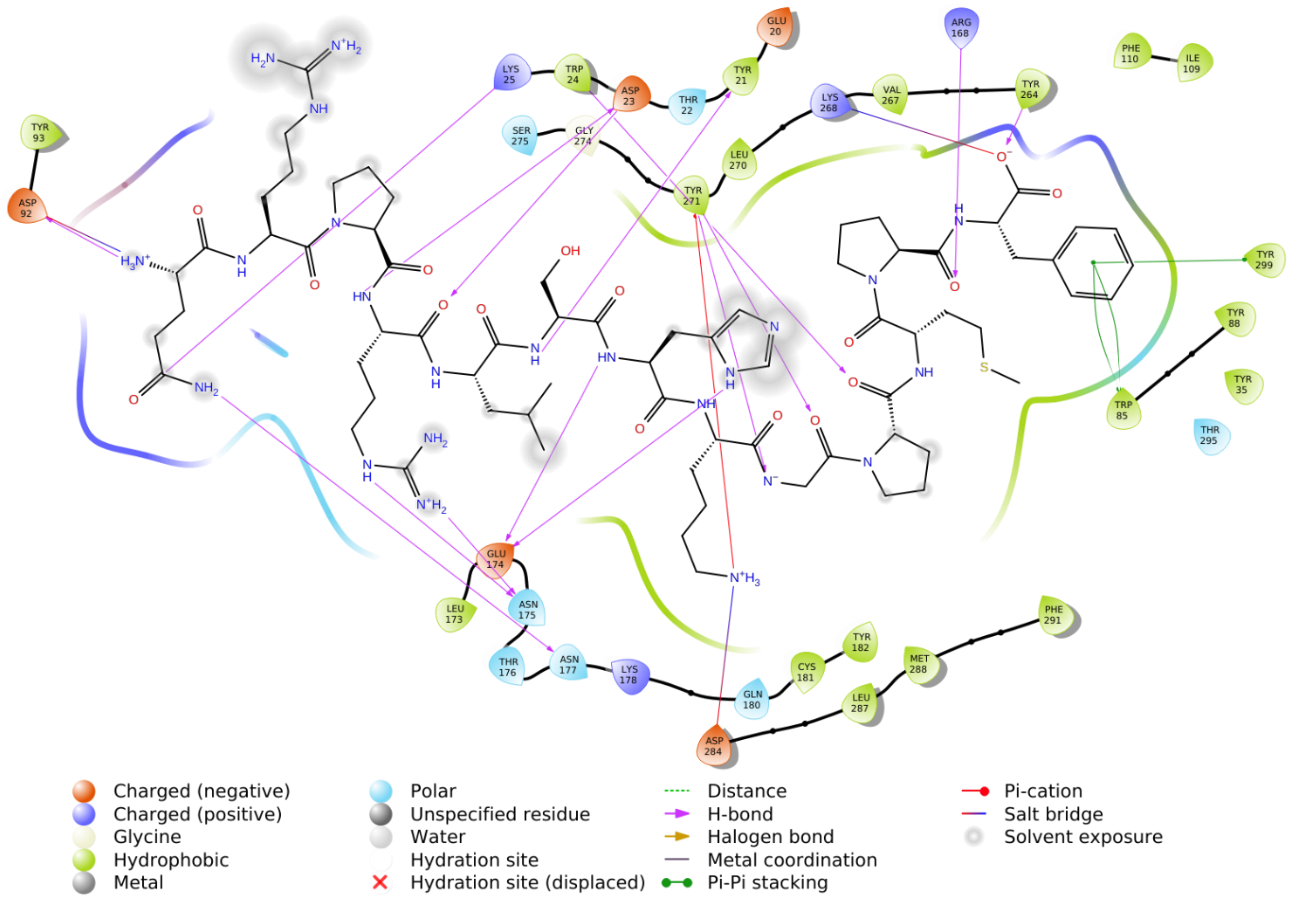

Fig. S8. Two-dimensional representation of the amino acid and apelin peptide interactions in our AlphaFold2 apelin receptor apelin peptide complex model.

Two-dimensional representation showing the principal interactions between the residues of [Pyr[1]]-apelin-13 and amino acids in the apelin receptor.

Residuals graphs for Fig 7a, apelin-13, ΔC2

Apelin-13

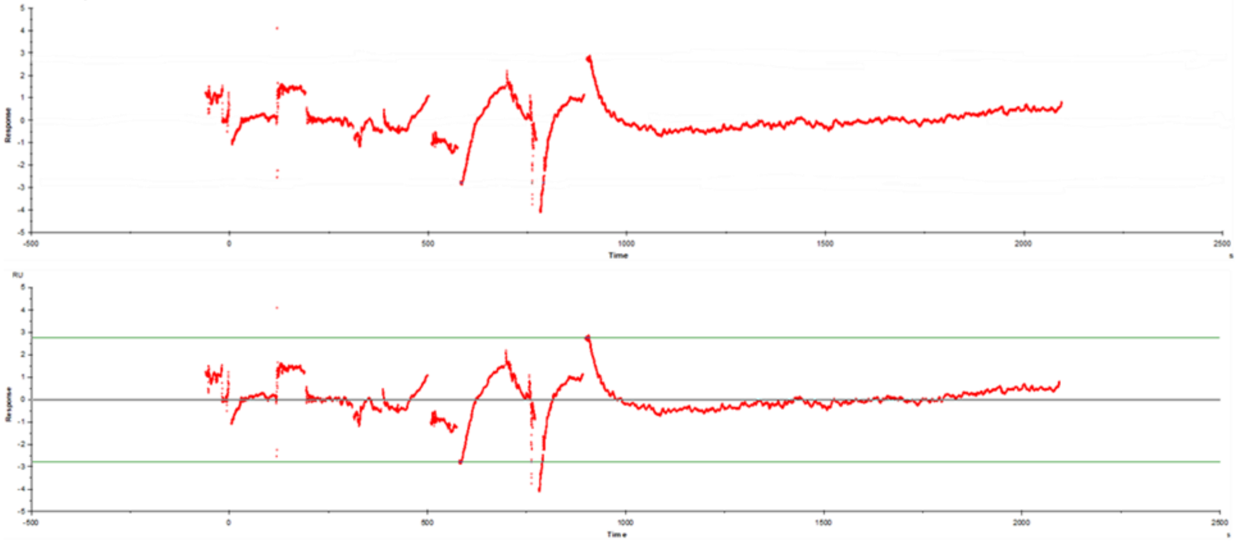

DeltaC2

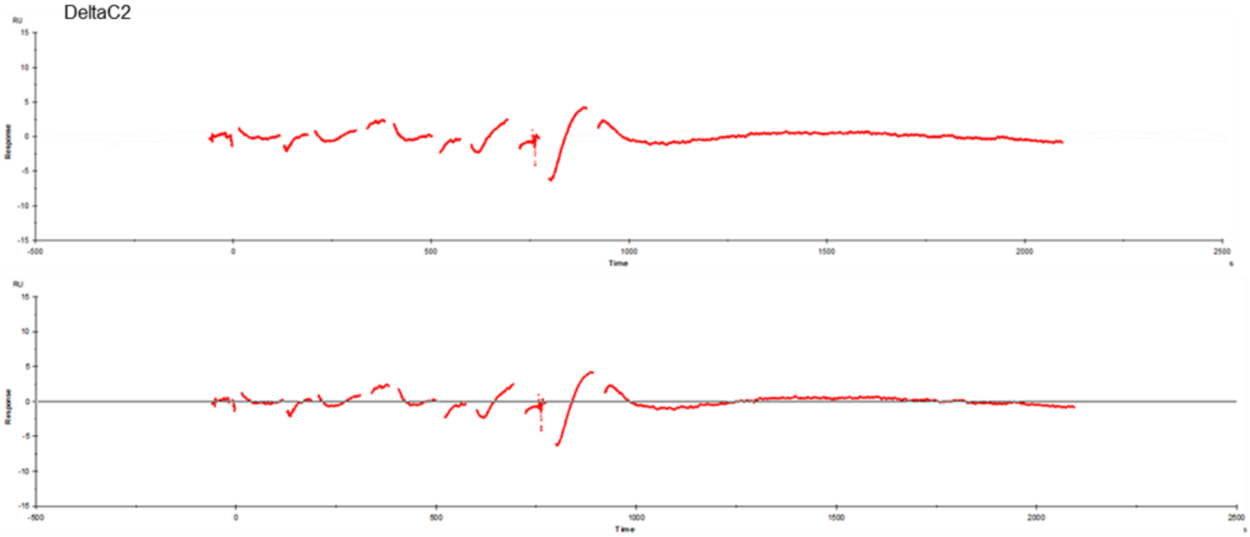

Residuals graphs for Fig 7b, upper panel, pH7.5

pH 7.5, StaR, Apelin-13

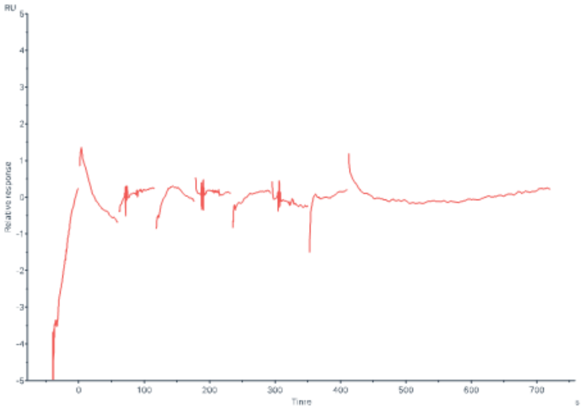

pH 7.5, R168H, Apelin-13

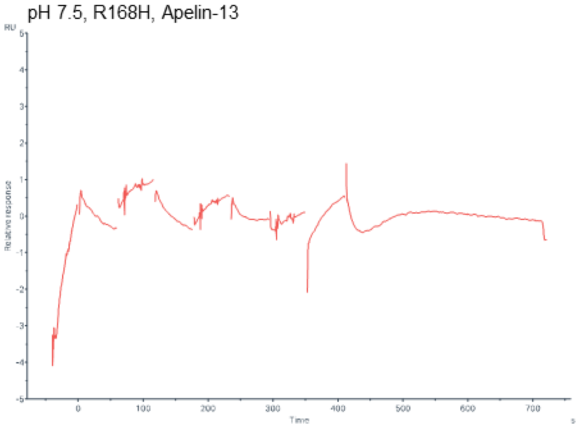

pH 7.5, StaR, NXE'992

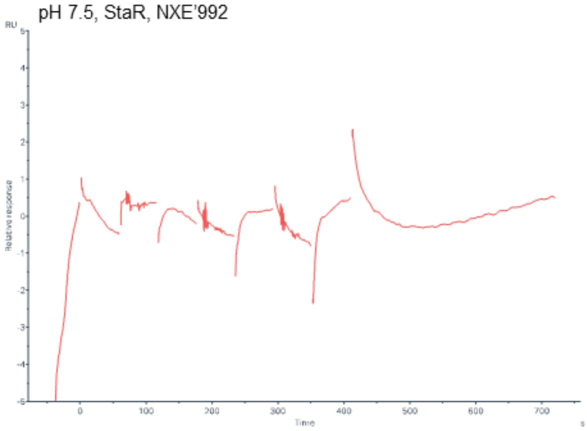

pH 7.5, R168H, NXE'992

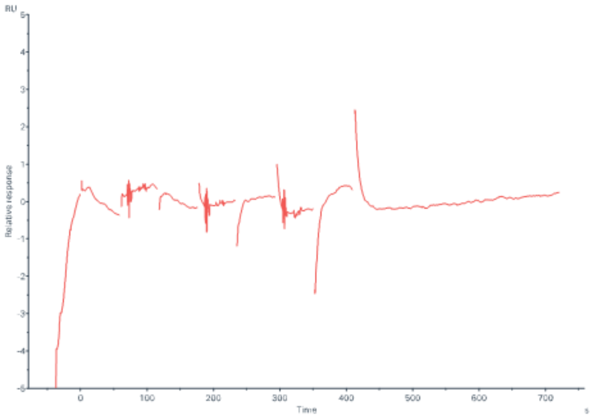

pH 7.5, StaR, CMF-019

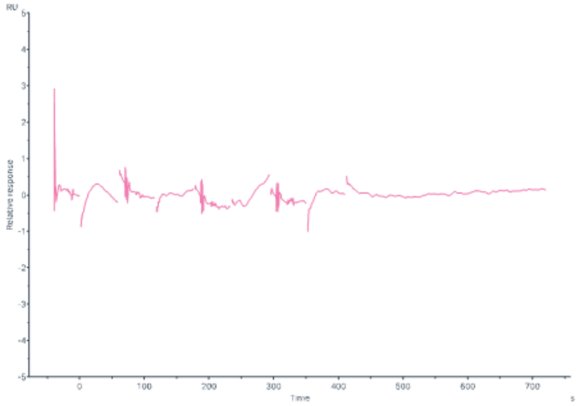

pH 7.5, R168H, CMF-019

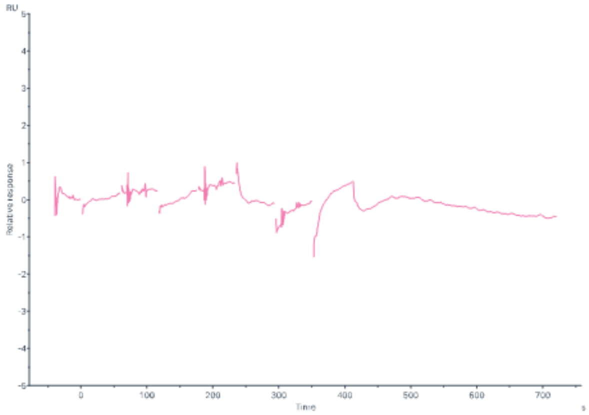

pH 7.5, StaR, NXE'870

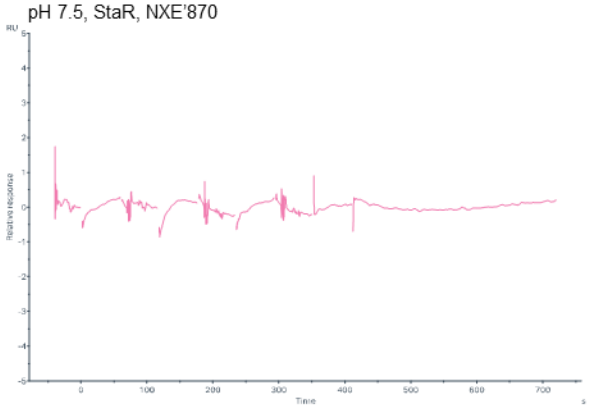

pH 7.5, R168H, NXE'870

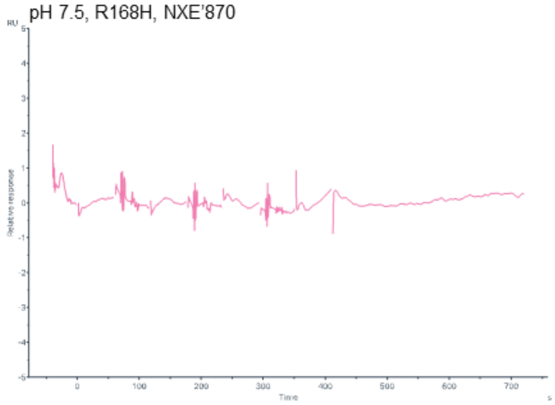

Residuals graphs for Fig 7b, lower panel, pH6.0

pH 6.0, StaR, Apelin-13

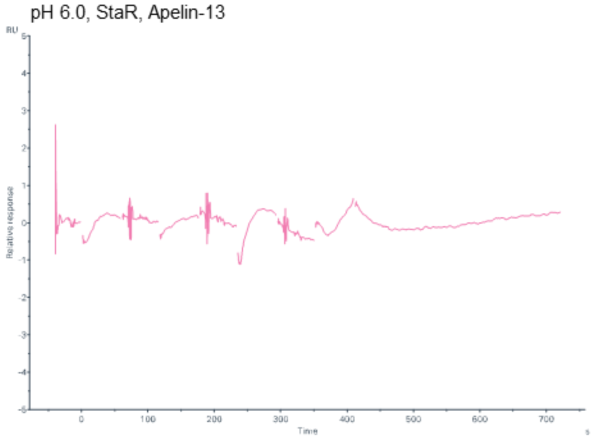

pH 6.0, R168H, Apelin-13

No Binding

pH 6.0, StaR, NXE'992

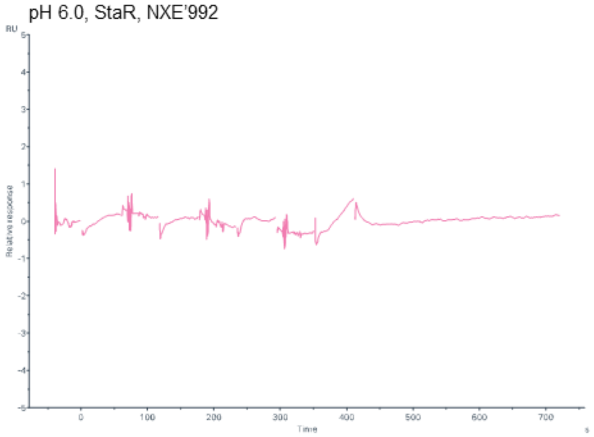

pH 6.0, R168H, NXE'992

No Binding

pH 6.0, StaR, CMF-019

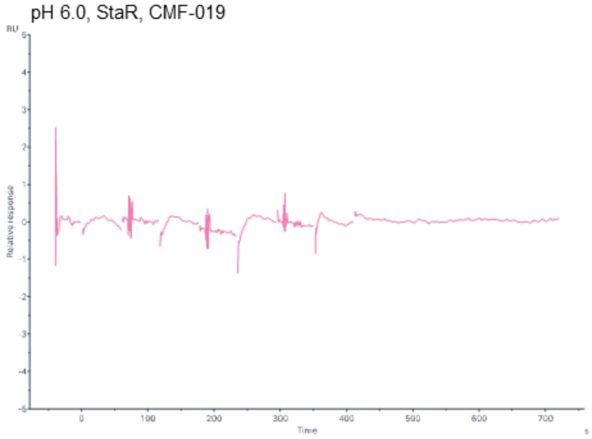

pH 6.0, R168H, CMF-019

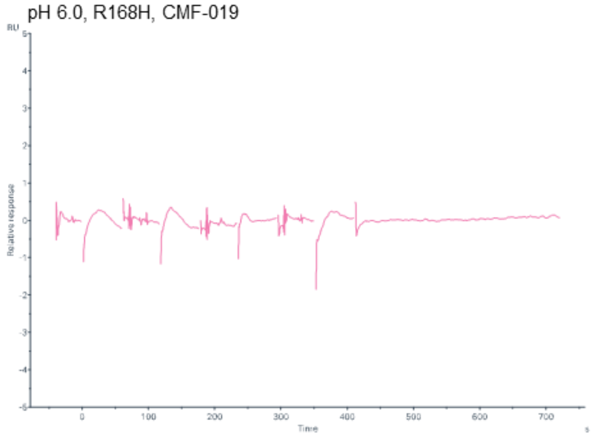

pH 6.0, StaR, NXE'870

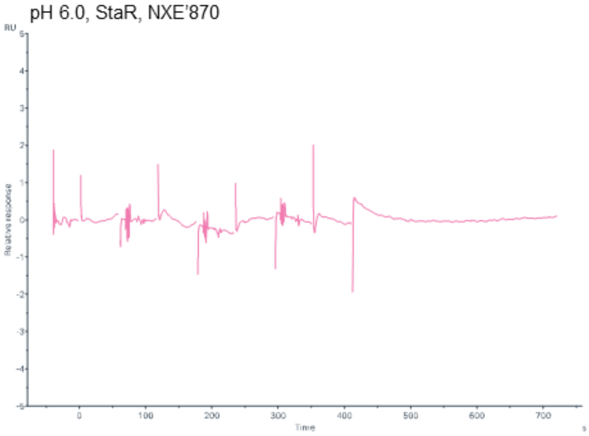

pH 6.0, R168H, NXE'870

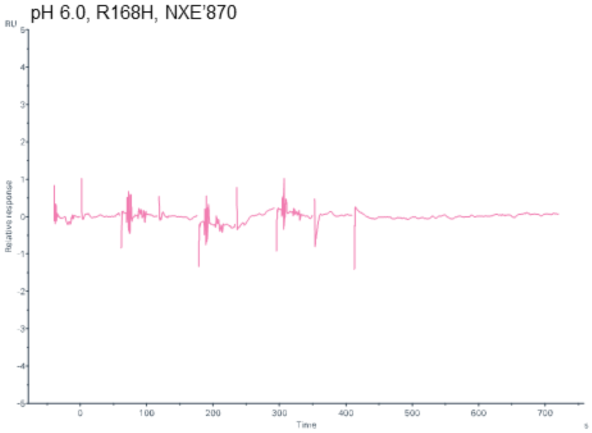

Fig. S9a: Residual plots for surface plasmon resonance sensorgrams of the apelin receptor NxStaR shown in Fig 7a and 7b, showing loss of binding of truncated peptides and that the R/H1684.64 variant can still bind small molecule agonists.

Residual plots showing the differences between fitted curves and measured responses for surface plasmon resonance sensorgrams shown for Fig 7a of apelin receptor NxStaR in the presence of endogenous apelin-13 peptide versus C-terminally truncated peptides (ΔC2).

Residual plots are shown for sensorgrams for 7b for apelin receptor NxStaR or the R/H1684.64 variant NxStaR at pH 7.5 in the presence of endogenous apelin-13 peptide, the chimeric peptide NXE’992, the G protein biased, small molecule agonist CMF-019 and the small synthetic agonist NXE’870.

Residual plots are shown for sensorgrams for 7b for apelin receptor NxStaR or the R/H1684.64 variant NxStaR at pH 6.0 in the presence of endogenous apelin-13 peptide, the chimeric peptide NXE’992, the G protein biased, small molecule agonist CMF-019 and the small synthetic agonist NXE’870. Source data are provided as a Source Data file.

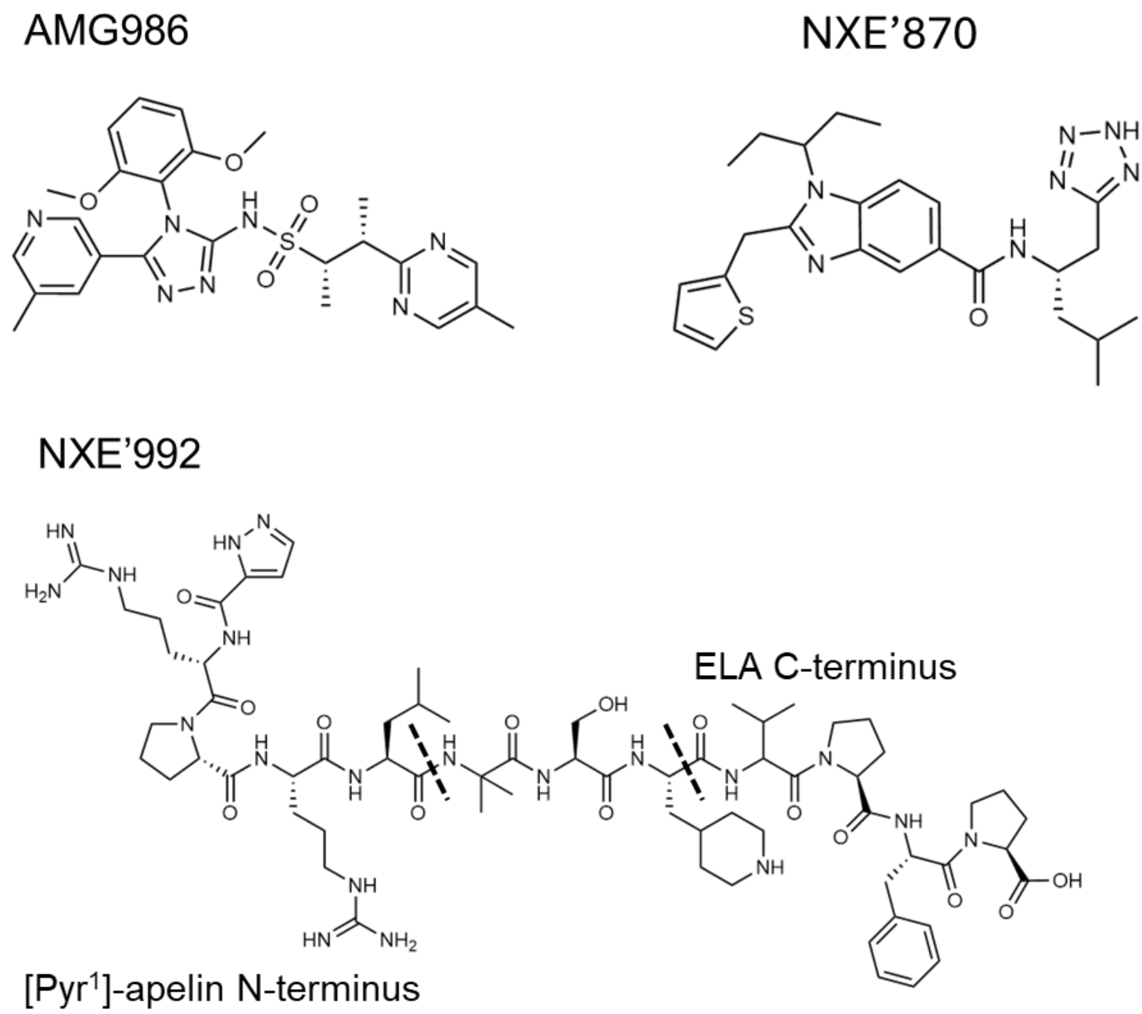

Fig. S9b: Chemical structures of the small synthetic agonists AMG-986 and NXE’870 BMS-986224 and of NXE’992, the chimera of the apelin and ELA peptides used in SPR experiments.

AMG 986 is a synthetic peptide agonist (Winkle, P. et al. A First-in-Human Study of AMG 986, a Novel Apelin Receptor Agonist, in Healthy Subjects and Heart Failure Patients. Cardiovascular Drugs and Therapy 37, 743-755 (2023). NXE’870 is a small synthetic agonist, compound 48 in Sanofi-Aventis patent #WO2014044738A (Hachtel, S. et al. Benzoimidazole-carboxylic acid amide derivatives as APJ receptor modulators. Google Patents, WO2014044738 A1 (2014). NXE’992 is a chimeric peptide that combines the five N-terminal residues of [Pyr[1]]apelin-13 peptide and the four C-terminal residues of ELA, linked by two unnatural amino acids and a serine ([Pyr]QRPRL[AIB]S[PipALA]VPFP). Chemical structures shown have been drawn using the Nature Chemistry template.

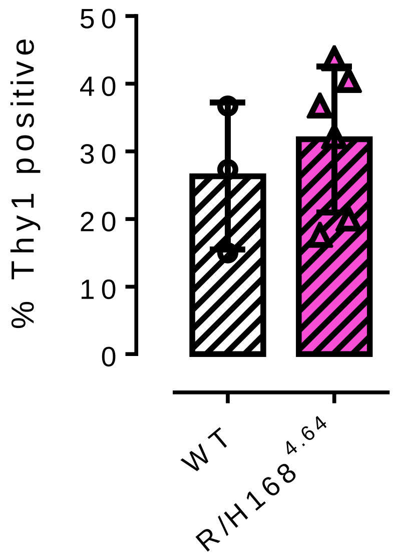

Fig. S10: Thy1 (fibroblast marker) expression in R/H168$^{4.64}$ variant hESC-CMs. Bar chart shows % hESC-CM population staining positive for the fibroblast marker Thy1 in wildtype (WT), n=3 mean±s.e.m, R/H168$^{4.64}$ n=3 independent experiments mean independent experiments ± s.e.m in duplicate). Source data are provided as a Source Data file.

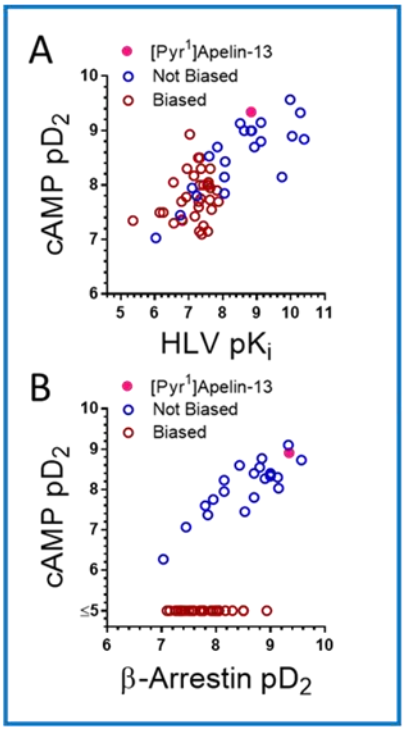

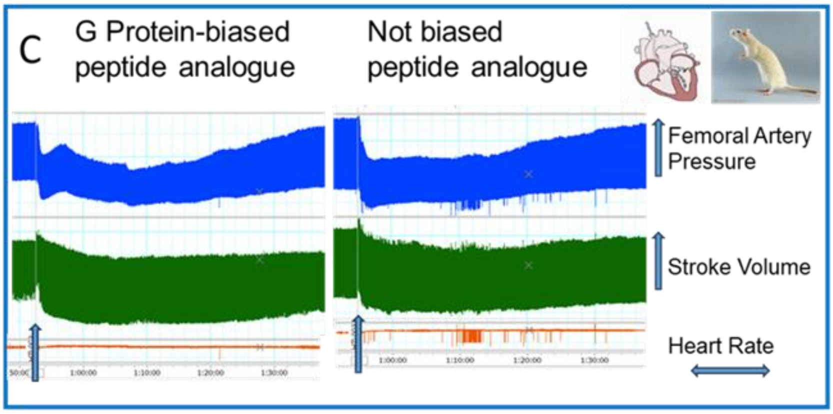

D

	cAMP pD_2	B-Arrestin pD_2	Rat in vivo $t_{1/2}$ (hrs)
G Protein Biased	8.9	<5	1.3
Not Biased	8.6	7.5	3.7

Fig. S11: Pharmacological characterisation of novel G protein-biased and not biased apelin receptor peptide agonists. a Over 50 peptide apelin receptor ligands were designed and synthesised that demonstrated high affinity in competition binding assays using [^{125}I]apelin-13 and sections of human left ventricle and were agonists in a G_i coupled cAMP assay in CHO-K1 cells expressing the human apelin receptor. **b** Using [Pyr^1]apelin-13 as the reference compound (solid magenta circle) compounds were either G protein biased (open red circles) or not biased (open blue circles) when tested in the G_i coupled cAMP assay and β-arrestin recruitment assay. **c** Both biased and not biased agonists produced the same desired short term beneficial cardiovascular actions when administered as a bolus, intravenously, in anaesthetised rats; increasing stroke volume and decreasing arterial pressure without increasing heart rate. These peptide agonists have much improved plasma half-lives compared to apelin. Source data are provided as a Source Data file.

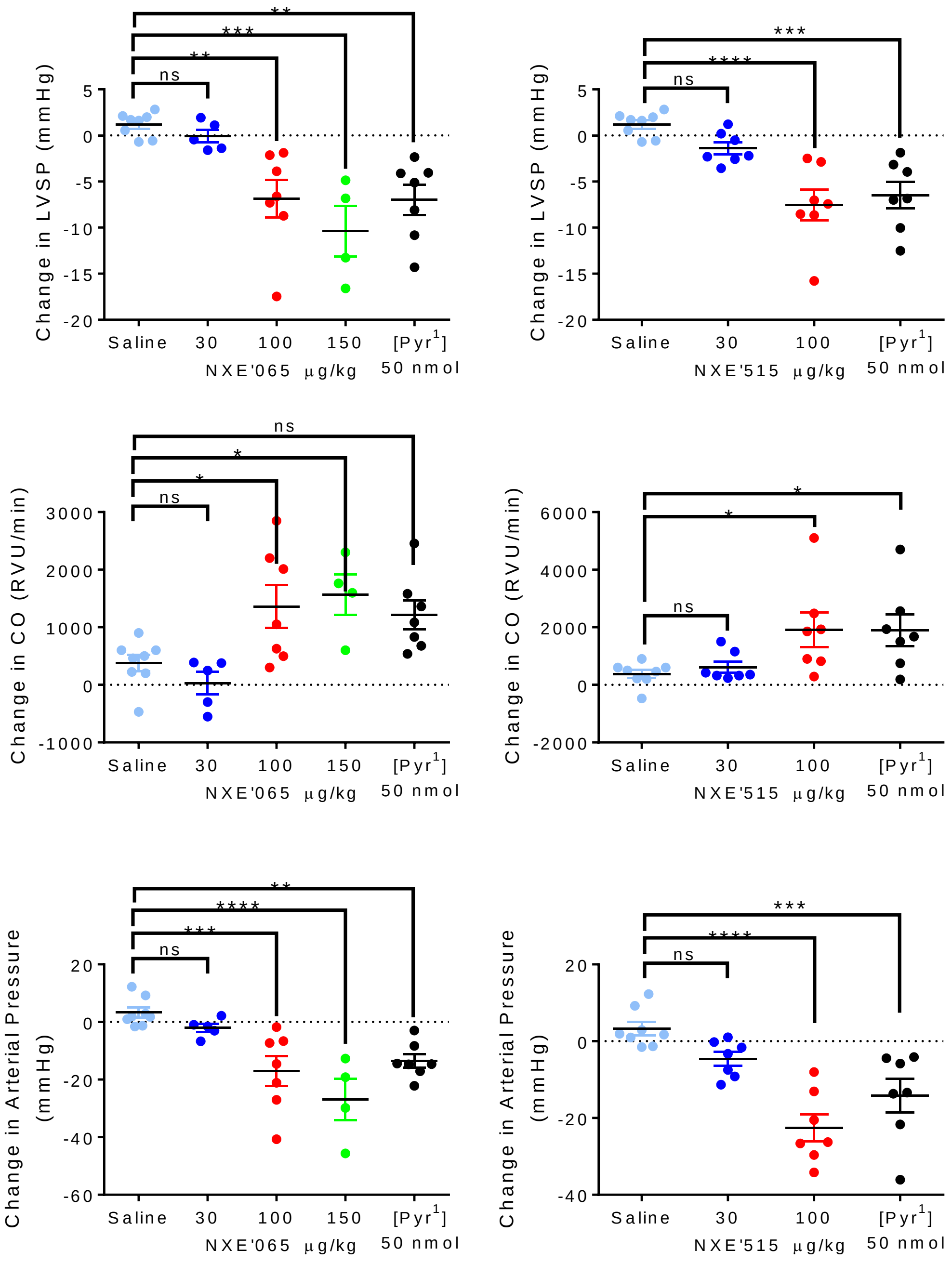

Fig. S12: Comparable i*n vivo* cardiovascular responses to a G protein-biased compound, NXE’065, and a not biased compound NXE’515 in anaesthetised rat.

Measurements of left ventricular systolic pressure (LVSP) and cardiac output(CO) were obtained via a pressure volume catheter placed in the left ventricle of the anaesthetised animals with change in arterial pressure measured via a pressure catheter placed in the femoral artery. Increasing bolus doses of either NXE’065 (30 (n=5), 100 (n=7) and 150 (n=4) µg/kg) or NXE’515 (30 (n=7) and 100 (n=7) µg/kg) and a reference bolus dose of 50 nmoles [Pyr^1]apelin-13 (n=7) were administered via the right external jugular vein (see Supplementary Methods below). The peak effects of NXE’065 or NXE’515 and [Pyr^1]apelin-13 on LVSP, CO and arterial pressure were compared to saline (n=8), expressed as change from baseline and compared by one-way ANOVA with Dunnett’s post-test. (* p<0.05; *** p<0.005; **** p<0.001 compared to saline). Both compounds produced a significant increase in CO and a significant decrease in LVSP and arterial pressure, a profile comparable to that of the reference peptide [Pyr^1]apelin-13. Source data are provided as a Source Data file.

Supplementary Methods:

Cardiovascular responses to a G protein-biased compound, NXE'065, and a non biased compound NXE'515 in anaesthetised rat.

All animal care and rodent experiments complied with the Home Office (United Kingdom) guidelines under the Animals (Scientific Procedures) Act 1986 Amendment Regulations (SI 2012/3,039) and were approved by the local ethics committee (University of Cambridge Animal Welfare and Ethical Review Body).

Male Sprague Dawley rats (300-400g, n=4-8 per group) were anaesthetised with gaseous isoflurane (3% for induction and 1.5-2% for maintenance, 1.5L/min oxygen). Body temperature was monitored using a rectal thermometer and maintained at 37°C. For compound administration, the right external jugular vein was cannulated with polyethylene tubing (Smiths Medical) filled with heparinised 0.9% saline solution. A pressure volume catheter (SPR-869, Millar, ADIstruments, Oxford, UK) was connected to the data PowerLab 16/35 system with LabChart 8 (ADIstruments, Oxford, UK) and calibrated using the MPVS Ultra PV Unit (ADIstruments, Oxford, UK). The catheter was inserted into the left ventricle (LV) via the right carotid artery. The position of the catheter was determined by the blood pressure and shape of the pressure volume loops. Animals was allowed to stabilise for recording of baseline hemodynamic parameters. A second catheter was inserted into the right femoral artery for the recording of arterial pressure at the same time as cardiac parameters were being measured. Increasing doses (2-3) of test peptides, (400µl bolus, peptide made up in 0.9% saline and administered at 30,100, 150 µg/kg) or the equal volume of saline were given as successive bolus intravenous injections via the cannula, which was flushed with 100µL saline after each injection. A bolus dose of [Pyr^1]apelin-13 (50nmoles) was administered after the test peptide doses. The effects of compounds were monitored for no less than 10 minutes. Low doses were monitored for peak effect (at ~1-2 mins after injection) and end effect at about 10 minutes. Higher doses showed a more protracted duration of action and therefore measurements were taken at about 1-2 mins, 10 mins and at the end of the recording period (approximately 30 mins) before administration of subsequent dose. Data analysis was performed using LabChart 8. The effects of test peptides, [Pyr^1]apelin-13 and saline on LV systolic pressure (LVSP), cardiac output (CO), and femoral artery pressure (AP) were expressed as change from baseline. Data (shown in Fig S11) are expressed as mean±sem. Data and statistical analyses were conducted in GraphPad Prism 6. P≤0.05 was considered statistically significant. and compared by one-way ANOVA with Dunnett's post-test

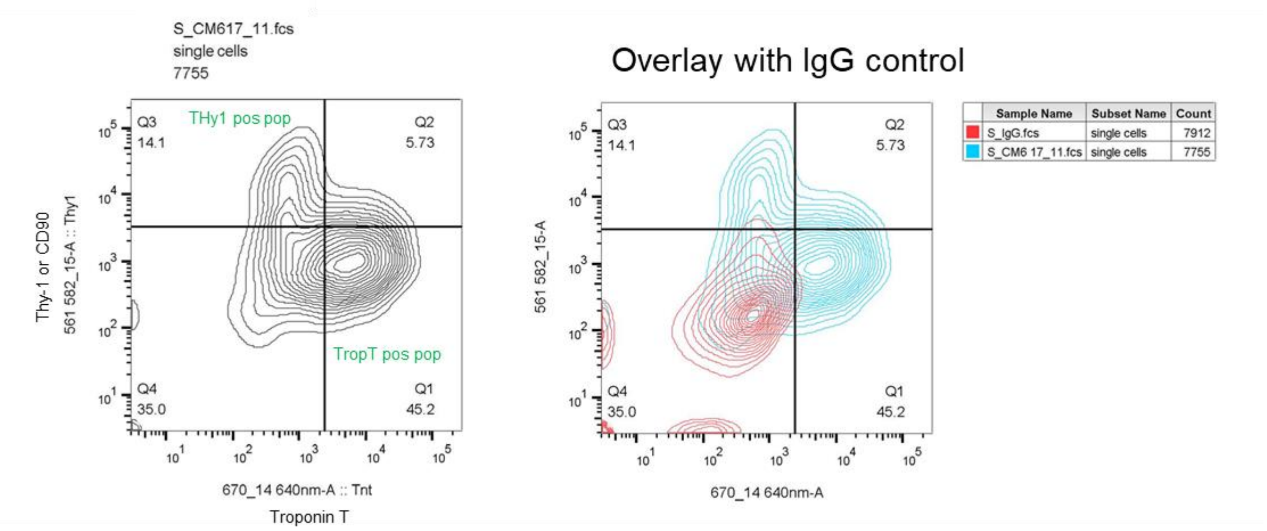

Fig. S13: Flow cytometry: gating strategy

Gating strategy: To ensure precise analysis of the cell population of interest, an unstained control was initially used to define the gate for viable (live) cells within the sample. Subsequently, doublet discrimination was performed to exclude doublets and aggregate events, isolating single cells for further analysis. This gating strategy was then applied to cells stained with an isotype control IgG to establish baseline fluorescence. Gates were carefully set based on the IgG control, with thresholds defined such that no more than 1% of the IgG-stained cells were positive for fluorescence signals detected at both 561 nm and 670 nm wavelengths. This conservative gating ensured minimal background signal contamination. The graph illustrates the gating strategy in detail. Cells stained for Troponin T (TropT) and Thy-1 (also known as CD90) are shown in blue, distinguishing two distinct populations: TropT-positive (Q1, Quarter 1) and Thy1-positive (Q3, Quarter 3) populations. The counterplot highlights the separation of these populations. For comparison, the IgG control is shown in red, providing a clear visual distinction between specific and non-specific staining.

Supplementary Methods:

The differentiation efficiency of hESCs harbouring the R/H168[4.64] variant into hESC-CMs was determined using flow cytometry. Populations of cardiomyocytes were harvested and pelleted by centrifugation at 300 xg for 3 mins. Pellets were resuspended in PBS supplemented with 0.1 % BSA and 2 mM EDTA (PBE), with CD90 (Thy-1) Monoclonal Antibody directly conjugated to PE (CD90 (Thy-1), from ThermoFisher, catalogue number,12-0909-42) diluted at 1:50, for 1 hour at 4 °C. Cells were then washed with PBE and resuspended in Fixation/Solubilization solution (BD Cytofix/Cytoperm Fixation/Permeabilization Kit, Biosciences) for 20 mins at 4 °C. Following incubation, cells were washed using 1X BD Perm/Wash Buffer (Biosciences) and then resuspended in 1X BD Perm/Wash Buffer containing directly conjugated Anti-Cardiac Troponin T-APC antibody(Miltenyi BioTech, catalogue number, 130-120-54300I) diluted at 1:50 and incubated for 2 hours at 4 °C. Cells were then washed in 1X BD Perm/Wash Buffer, resuspended in PBE and transferred to flow tubes. Samples were run on the LSRFortessa Cell Analyzer (BD Biosciences, https://www.bdbiosciences.com/en-us) and analysis performed using FlowJo v10.8.1 software (https://www.flowjo.com/solutions/flowjo/). 80 ± 9 % of wild-type hESC-CM cells stained positive for the cardiac specific marker troponin T versus Thy-1 positive cells similar to previous studies (Macrae et al., 2021 https://doi.org/10.1093/cvr/cvac065), indicating high purity.

Supplementary Tables

Table S1: Summary of apelin receptor residues that interact with the small synthetic agonists CMF-019 and cmpd644, and the endogenous peptides, apelin and ELA.

	$Y35^{1.39}$	$F78^{2.53}$	$L82^{2.57}$	$W85^{2.60}$	$Y88^{2.63}$	$S106^{3.29}$	$I109^{3.32}$	$F110^{3.33}$	$M113^{3.36}$	$R168^{4.64}$	$Y185^{45.54}$	$W261^{6.48}$	$Y264^{6.51}$	$V267^{6.54}$	$K268^{6.55}$	$Y271^{6.58}$	$F291^{7.35}$	$P292^{7.35}$	$T295^{7.39}$	$S/A298^{7.42}$	$Y299^{7.43}$
CMF-019	III	III	II	II		II	II/III	III	II	II	ECV	III	III	I	I	I	I		II/III	III	II/III
cmpd644		III		II/III	II	II	II/III	III	II	II		III	III	I	I	I	I		III	III	III
apelin				·	·		·	·		·			·			·			·		·
ELA				·	·		·	·		·			·			·		·	·		·

Ligand	Receptor	pH	ka (M-1s-1)	kd (s-1)	KD (M)
apelin-13	StaR	7.5	2.3E+06	7.2E-04	3.1E-10
		6.0	2.4E+06	6.2E-03	2.6E-09
	StaR R168H	7.5	7.6E-05	2.5E-02	3.4E-08
		6.0	N/A	N/A	no binding
NXE’992	StaR	7.5	8.0E+05	2.6E-03	3.3E-09
		6.0	5.3E+05	1.1E-02	2.1E-08
	StaR R168H	7.5	N/A	N/A	no binding
		6.0	N/A	N/A	no binding
NXE’870	StaR	7.5	1.0E+06	1.9E-03	1.9E-09
		6.0	1.8E+06	1.3E-03	7.2E-10
	StaR R168H	7.5	7.1E+05	2.7E-02	3.8E-08
		6.0	2.6E+06	9.6E-03	3.8E-09
CMF-019	StaR	7.5	1.3E+06	7.8E-03	6.1E-09
		6.0	1.8E+06	6.0E-03	3.4E-09
	StaR R168H	7.5	4.1E+05	2.8E-02	6.9E-08
		6.0	2.3E+06	3.3E-02	1.5E-08

Table S2: Summary table of SPR data for the apelin receptor NxStaR and R168H variant binding to apelin, chimeric peptide NXE’992, investigational ligand NXE’870 and CMF-019, tested at pH 7.5 and 6.0. Each value represents a single determination.

NxStaR mutation	GFP (% WT)	B_{max} (4 °C)	Ratio	Ratio - WT	B_{max} (% WT)	Stability index
T/V87$^{2.62}$	139.68	483.00	0.71	0.28	99.26	1.28
N/A112$^{3.35}$	78.48	705.50	0.77	0.24	121.49	1.24
T/M207$^{5.44}$	118.43	470.50	0.27	-0.08	122.43	0.92
F/L214$^{5.51}$	90.37	408.50	0.65	0.10	108.28	1.10
I/A224$^{5.61}$	89.08	1409.00	0.48	-0.07	373.49	0.93
S/A298$^{7.42}$	99.94	3119.00	0.80	0.27	248.23	1.31

Table S3. Summary of the impact of the selected apelin stabilised receptor (NxStaR) mutations on expression, radioligand binding, and thermostability. Overall expression was determined using GFP signal detected by PHERANxStaR microplate reader and reported as a percentage of the wild-type (WT) template. Receptor density (B_{max}) was determined in the presence of the radioligand [^{3}H]-NXE'870 at 4 °C. Thermostability was measured by incubation at different temperatures and expressed as a ratio (B_{max} at 4 °C / B_{max} at higher temperature). B_{max} was also expressed as a percentage of the WT template. Importantly, a stability index (1 + (Ratio - WT)) greater than 1 is classed as stabilising versus the WT template. Each value represents a single determination.

	NxStaR-CMF-019
**Data collection**	
Number of crystals	7
Space group	$C222_1$
Cell dimensions	
a, *b*, *c* (Å)	65.82, 153.95, 111.65
α, β, γ (°)	90, 90, 90
Resolution (Å)	76.97-2.58 (3.03-2.58)*
No. reflections	9698 (223)*
R_{pim} (all I^+ & I^-)	0.029 (0.145)*
$I/\sigma(I)$	7.1 (1.5)*
$CC_{1/2}$	0.923 (0.497)*
Completeness (%)	
spherical	54.5 (4.3)*
ellipsoidal	90.1 (38.6)*
Redundancy	14.8 (18.4)*
**Refinement**	
Resolution (Å)	76.97-2.58
R_{work} / R_{free}	0.224/0.278
No. atoms	
Protein	3016
Ligand	32
Other	76
B factors	
Protein	66.75
Ligand	45.56
Other	43.65
R.m.s. deviations	
Bond lengths (Å)	0.01
Bond angles (°)	1.05

* Values in parentheses are the for highest-resolution shell.

Table S4: Crystallographic X-ray data collection and structure refinement statistics.

Compound ID	Cell Based Assays		Human Heart Homogenate
	cAMP (pD_2)±SD (n)	β-Arrestin (pD_2)±SD (n)	pK_i±sem (n=3)
Compounds that are not G protein biased			
NXE'686	8.8± 0.1 (3)	8.77±0.21 (3)	10.39±0.02
NXE'689	7.85 (2)	7.37±0.12 (3)	8.05±0.03
NXE'996	8.15 (2)	7.95 (2)	8.05±0.08
NXE'099	8.70±0.36 (3)	7.80±0.17 (3)	7.84±0.06
NXE'403	9.00±0.50 (3)	8.37±0.25 (3)	8.82±0.20
NXE'485	7.80±0.26 (3)	7.60±0.35 (3)	7.24±0.02
NXE'506	9.15 (2)	8.03±0.21 (3)	9.12±0.06
NXE'507	8.90±0.10(3)	8.27±0.32 (3)	10.04±0.08
NXE'987	7.45 (2)	7.07±0.38(3)	6.75±0.03
NXE'998	8.43±0.32 (3)	8.60±0.61 (3)	8.07±0.39
NXE'456	8.8 (1)	8.55 (1)	9.12±0.47
NXE'458	8.15 (2)	8.25±0.07 (2)	9.74±0.20
NXE'461	8.70±0.14 (2)	8.40 (2)	8.93±0.11
NXE'520	9.57±0.38 (3)	8.73±0.31 (3)	9.98±0.18
NXE'522	9.33±0.12 (3)	9.10±0.20 (3)	10.28±0.25
NXE'810	7.03±0.31 (3)	6.27±0.31 (3)	6.03±0.03
NXE'924	9.0±0.26 (3)	8.33±0.06 (3)	8.63±0.06
NXE'181	7.95(2)	7.75 (2)	7.10±0.02
NXE'709	9.0±0.36 (3)	8.4±0.10 (3)	8.85±0.05
NXE'712	9.13±0.21 (3)	8.53±0.50 (3)	8.51±0.10
NXE'515	8.53±0.23 (3)	7.45 (2)	7.60±0.12
Compounds that are G protein biased			
NXE'481	7.43±0.49 (3)	<5 (3)	7.18±0.03
NXE'482	7.73±0.06 (3)	<5 (3)	7.62±0.10
NXE'488	7.1 (2)	<5 (2)	7.38±0.03
NXE'490	7.15 (2)	<5 (2)	7.56±0.01
NXE'524	7.90±0.10 (3)	<5 (3)	7.83±0.06
NXE'525	8.17±0.12 (3)	<5 (3)	7.15±0.01
NXE'526	7.78 (2)	<5 (2)	6.93±0.04
NXE'670	8.05 (2)	<5 (2)	7.58±0.03
NXE'674	7.75 (2)	<5 (2)	7.35±0.05
NXE'677	7.15 (2)	<5 (2)	7.31±0.05
NXE'678	7.95 (2)	<5 (2)	7.63±0.02
NXE'679	7.50±0.53 (3)	<5 (3)	6.25±0.05
NXE'681	7.50 (2)	<5 (2)	6.15±0.04
NXE'697	8.0±0.1 (3)	<5 (5)	7.43±0.03
NXE'710	7.70±0.30(3)	<5 (3)	7.87±0.06
NXE'799	7.25±0.07 (3)	<5 (3)	7.43±0.05
NXE'801	7.37±0.15 (3)	<5 (3)	6.80±0.07
NXE'062	8.5±0 (3)	<5 (3)	7.28±0.14
NXE'063	8.0±0.44 (3)	<5 (3)	7.58±0.04

NXE’065	8.93±0.15 (3)	<5 (3)	7.03±0.02
NXE’068	8.5 (2)	<5 (2)	7.33±0.09
NXE’510	8.05 (2)	<5 (2)	6.55±0.03
NXE’512	8.3 (2)	<5 (2)	6.95±0.11
NXE’526	7.3 (2)	<5 (2)	6.56±0.04
NXE’531	7.35 (2)	<5 (2)	6.81±0.04
NXE’532	7.60 (2)	<5 (2)	7.29±0.03
NXE’533	7.7 (2)	<5 (2)	7.29±0.14
NXE’534	7.55 (2)	<5 (2)	7.67±0.06
NXE’776	8.0 (2)	<4 (2)	7.32±0.03
NXE’779	8.30 (2)	<4 (2)	7.63±0.02
NXE’855	7.35 (2)	<4 (1)	5.36±0.01
NXE’351	7.70 (2)	<4 (2)	6.79±0.04
NXE’354	8.0 (2)	<4 (2)	7.54±0.10
NXE’355	8.30(2)	<4 (2)	7.36±0.01

Table S5: Potency (pEC_{50}) of novel apelin peptide agonists in a cAMP and β-arrestin recruitment assays in cells expressing the human apelin receptor and binding affinity (pK_i) of these compounds in human heart (left ventricle) homogenate binding assay.

Compounds were determined as not biased if they demonstrated appreciable potency in the two cell-based assays and as G protein-biased if they inhibited forskolin stimulated cAMP accumulation (apelin is a Gi coupled receptor) but demonstrated no agonist activity in the β-arrestin recruitment assay at concentrations of up to 10-100μM.
pD_2 = -log10 of the EC50 value (where the EC50 is the molar concentration of the test compound producing half maximal response).
Each pD_2 value derived from the concentration response curves of both the cAMP and β-arrestin recruitment assays are presented as mean ± SD where n is >2 and, as mean only where n=2. n values are given in parentheses
pKi = -log10 Ki value (where the Ki is the concentration of test compound competing for 50% of the specific binding of the radiolabelled compound [125I]apelin-13 (0.1nM).
Data for heart homogenate radioligand binding assays are expressed as mean ± s.e.m., n = 3 independent experiments. Source data are provided as a Source Data file.

Supplementary Methods:

Cell based assays. Activation of β-Arrestin by apelin ligands was measured using the *in vitro* The PathHunter® CHO-K1 AGTRL1 β-Arrestin Cell Line (Eurofins, Catalog # 93-1050E2CP2M) expressing the apelin receptor. Cells were plated in a 384-well plate and incubated overnight at 37°C and 5% CO2 to allow the cells to attach and grow. Compounds were incubated for 1.5 h together with [Pyr^1]-apelin 13 as a reference and β-Arrestin accumulation detected using the PathHunter Detection Kit according to the recommended protocol (https://cf-ecom-products.discoverx.com/download/Products/UserManual/eXpressKits/70-255_PathHunter_B-Arrestin_eXpress_GPCR_Assay.pdf).

Inhibition of cAMP signalling by cells expressing the apelin receptor was determined using the cAMP Hunter™ eXpress AGTRL1 CHO-K1 GPCR Assay (Eurofins, Catalog # 95-0147E2CP2M). Compounds were incubated for 1.5

h together with [Pyr^1]-apelin 13 as a reference and cAMP inhibition measured cAMP Hunter eXpress according to the recommended protocol (https://cf-ecom-products.discoverx.com/download/Products/UserManual/eXpressKits/70-234_cAMP_Hunter_eXpress_GPCR_Assay.pdf).

Heart Homogenate. Ethically approved (Research Ethics Committee 05/Q104/142) surgical samples of human left ventricle tissue were obtained with informed consent at the time of operation from Royal Papworth Hospital Tissue Bank and stored at -80°C. Tissues were homogenised using a Polytron Homogenizer (Thomas Scientific), in buffer (50 mM Tris-HCl, 5 mM MgCl2, 5 mM EDTA, 1 mM EGTA, 1:500 protease inhibitor cocktail containing aprotinin and amastatin, balanced at pH 7.4) at 4°C. The homogenate was then spun at 1000 xg for 2 mins at 4°C and the supernatant centrifuged at 40,000 xg, for 30 mins at 4°C. The pellet was resuspended in 2.5 mL/g of homogenisation buffer, followed by a second spin at 40,000 xg, for 30 mins at 4°C. The resulting pellet was resuspended in HEPES buffer (50 mM HEPES, balanced at pH 7.4 at room temperature) and stored in the freezer at -70°C until use in assays.

Competition binding assay were performed using 0.1 nM [Glp65,Nle75,Tyr77][125I]-apelin-13 (PerkinElmer, NEX393, specific activity of 2200 Ci/mmol.). Increasing concentrations of test ligand were incubated over a concentration range (1 pM – 1 µM) with the ligand in binding buffer (50 mM TRIS-HCl, 5 mM MgCl2, pH 7.4) for 90 mins at room temperature. To terminate equilibrium, samples were centrifuged at 20,000 xg for 10 mins at 4°C. Pellets were washed by resuspending 500 µL ice-cold wash buffer (50 mM TRIS-HCl, pH 7.4) and centrifuged a second time at 20,000 xg for 10 mins at 4°C, before aspiration of the supernatant.

Radioactivity in pellets was counted using a Cobra II model 5003 gamma counter (Packard). Competition binding data were analysed using the nonlinear one site Fit Ki model using GraphPad Prism version 6.07, with constraints for the radiolabel concentration (0.1 nM) and KD (0.076 nM, previously determined by saturation analysis in homogenised human left ventricle) input. The software was also used to calculate binding affinities (Ki values) of competing ligands using the Cheng-Prusoff equation.
