# Supplementary material for: Structural and functional determination of peptide versus small molecule ligand binding at the apelin receptor
Source: Nat Commun. 2024 Dec 27;15:10714. doi: 10.1038/s41467-024-55381-w (PMC11680790; doi:10.1038/s41467-024-55381-w)
Supplement: Supplementary file 2 — Reporting Summary [file 41467_2024_55381_MOESM2_ESM.pdf]

nature portfolio

Corresponding author(s): Professor Anthony Davenport, Dr Alastair Brown

Last updated by author(s): Jun 14, 2024

Reporting Summary

Nature Portfolio wishes to improve the reproducibility of the work that we publish. This form provides structure for consistency and transparency in reporting. For further information on Nature Portfolio policies, see our Editorial Policies and the Editorial Policy Checklist.

Statistics

For all statistical analyses, confirm that the following items are present in the figure legend, table legend, main text, or Methods section.

n/a	Confirmed	
☐	☒	The exact sample size (*n*) for each experimental group/condition, given as a discrete number and unit of measurement
☐	☒	A statement on whether measurements were taken from distinct samples or whether the same sample was measured repeatedly
☐	☒	The statistical test(s) used AND whether they are one- or two-sided<br>*Only common tests should be described solely by name; describe more complex techniques in the Methods section.*
☒	☐	A description of all covariates tested
☐	☒	A description of any assumptions or corrections, such as tests of normality and adjustment for multiple comparisons
☐	☒	A full description of the statistical parameters including central tendency (e.g. means) or other basic estimates (e.g. regression coefficient) AND variation (e.g. standard deviation) or associated estimates of uncertainty (e.g. confidence intervals)
☐	☒	For null hypothesis testing, the test statistic (e.g. *F*, *t*, *r*) with confidence intervals, effect sizes, degrees of freedom and *P* value noted<br>*Give P values as exact values whenever suitable.*
☒	☐	For Bayesian analysis, information on the choice of priors and Markov chain Monte Carlo settings
☒	☐	For hierarchical and complex designs, identification of the appropriate level for tests and full reporting of outcomes
☒	☐	Estimates of effect sizes (e.g. Cohen's *d*, Pearson's *r*), indicating how they were calculated

Our web collection on statistics for biologists contains articles on many of the points above.

Software and code

Policy information about availability of computer code

Data collection	N/A
Data analysis	N/A

For manuscripts utilizing custom algorithms or software that are central to the research but not yet described in published literature, software must be made available to editors and reviewers. We strongly encourage code deposition in a community repository (e.g. GitHub). See the Nature Portfolio guidelines for submitting code & software for further information.

Data

Policy information about availability of data

All manuscripts must include a data availability statement. This statement should provide the following information, where applicable:

- Accession codes, unique identifiers, or web links for publicly available datasets
- A description of any restrictions on data availability
- For clinical datasets or third party data, please ensure that the statement adheres to our policy

Source data are provided with this data as Source Data file. The crystal structure of the stabilized Apelin receptor (StaR) and in complex with CMF-019 described in this study has been deposited at the Protein Data Bank (PDB) under the accession code: PDB ID 8S4D (PDB DOI: https://doi.org/10.2210/pdb8S4D/pdb).

Research involving human participants, their data, or biological material

Policy information about studies with human participants or human data. See also policy information about sex, gender (identity/presentation), and sexual orientation and race, ethnicity and racism.

Reporting on sex and gender	N/A
Reporting on race, ethnicity, or other socially relevant groupings	N/A
Population characteristics	N/A
Recruitment	N/A
Ethics oversight	N/A

Note that full information on the approval of the study protocol must also be provided in the manuscript.

Field-specific reporting

Please select the one below that is the best fit for your research. If you are not sure, read the appropriate sections before making your selection.

[x] Life sciences   [ ] Behavioural & social sciences   [ ] Ecological, evolutionary & environmental sciences

For a reference copy of the document with all sections, see nature.com/documents/nr-reporting-summary-flat.pdf

Life sciences study design

All studies must disclose on these points even when the disclosure is negative.

Sample size	Sample sizes are indicated in the text.
Data exclusions	No data were excluded.
Replication	Biological experiments had at least three separate replicates.
Randomization	N/A
Blinding	N/A

Reporting for specific materials, systems and methods

We require information from authors about some types of materials, experimental systems and methods used in many studies. Here, indicate whether each material, system or method listed is relevant to your study. If you are not sure if a list item applies to your research, read the appropriate section before selecting a response.

Materials & experimental systems

n/a	Involved in the study	
[ ]	[x]	Antibodies
[ ]	[x]	Eukaryotic cell lines
[x]	[ ]	Palaeontology and archaeology
[ ]	[x]	Animals and other organisms
[x]	[ ]	Clinical data
[x]	[ ]	Dual use research of concern
[x]	[ ]	Plants

Methods

n/a	Involved in the study	
[x]	[ ]	ChIP-seq
[ ]	[x]	Flow cytometry
[x]	[ ]	MRI-based neuroimaging

Antibodies

Antibodies used	Primary antibodies: apelin receptor antibody (Sigma-Aldrich, SAB2700205, 1:50); Anti-Cardiac Troponin T-APC (Miltenyi BioTech, 130-120-54300I)1:50); CD90 (Thy-1) Monoclonal Antibody (eBio5E10 (5E10)1:50), PE, eBioscience (ThermoFisher, 12-0909-42). Secondary antibody: Donkey Anti-Rabbit IgG H&L (Alexa Fluor 488) (abcam, ab150073, 1:200)
Validation	Web links for antibodies used in this study:

apelin receptor antibody (Sigma-Aldrich, SAB2700205, 1:50) https://www.citeab.com/antibodies/1488138-sab2700205-anti-aplnr-n-terminal-antibody-produced?des=f526adee082ba529
Anti-Cardiac Troponin T-APC (Miltenyi BioTech, 130-120-54300) https://www.miltenyibiotec.com/GB-en/products/cardiac-troponin-t-antibody-anti-human-mouse-rat-reafinity-rea400.html#conjugate=apc:size=100-tests-in-200-ul
CD90 (Thy-1) Monoclonal Antibody (eBio5E10 (5E10)), PE, eBioscience (ThermoFisher, 12-0909-42) https://www.thermofisher.com/antibody/product/CD90-Thy-1-Antibody-clone-eBio5E10-5E10-Monoclonal/12-0909-42
Donkey Anti-Rabbit IgG H&L (Alexa Fluor 488) (abcam, ab150073, 1:200) https://www.abcam.com/products/secondary-antibodies/donkey-rabbit-igg-hl-alexa-fluor-488-ab150073.html

Eukaryotic cell lines

Policy information about cell lines and Sex and Gender in Research

Cell line source(s)	CHO-K1 were sourced from the European Collection of Cell Cultures (ECACC, 85051005). H9 human embryonic stem cells were obtained from Wicell Stem Cell Bank, Wisconsin, USA. WA09 (H9) hESCS - female, normal karyotype
Authentication	Normal karyotype confirmed, no other authentication
Mycoplasma contamination	Regularly tested negative for mycoplasma
Commonly misidentified lines (See ICLAC register)	No commonly misidentified cell lines were used in the study.

Animals and other research organisms

Policy information about studies involving animals; ARRIVE guidelines recommended for reporting animal research, and Sex and Gender in Research

Laboratory animals	Male Sprague Dawley rats (300-400g, n=4-8 per group)
Wild animals	n/a
Reporting on sex	This was a short term acute study in males only, measuring the action of bolus intravenous injections via a cannula, within 30 min on the cardiovascular system rather than an vivo study lasting for several days, where sex differences may be important.
Field-collected samples	n/a
Ethics oversight	All animal care and rodent experiments complied with the Home Office (United Kingdom) guidelines under the Animals (Scientific Procedures) Act 1986 Amendment Regulations (SI 2012/3,039) and were approved by the local ethics committee (University of Cambridge Animal Welfare and Ethical Review Body).

Note that full information on the approval of the study protocol must also be provided in the manuscript.

Plants

Seed stocks	*Report on the source of all seed stocks or other plant material used. If applicable, state the seed stock centre and catalogue number. If plant specimens were collected from the field, describe the collection location, date and sampling procedures.*
Novel plant genotypes	*Describe the methods by which all novel plant genotypes were produced. This includes those generated by transgenic approaches, gene editing, chemical/radiation-based mutagenesis and hybridization. For transgenic lines, describe the transformation method, the number of independent lines analyzed and the generation upon which experiments were performed. For gene-edited lines, describe the editor used, the endogenous sequence targeted for editing, the targeting guide RNA sequence (if applicable) and how the editor was applied.*
Authentication	*Describe any authentication procedures for each seed stock used or novel genotype generated. Describe any experiments used to assess the effect of a mutation and, where applicable, how potential secondary effects (e.g. second site T-DNA insertions, mosiacism, off-target gene editing) were examined.*

Flow Cytometry

Plots

Confirm that:

- [x] The axis labels state the marker and fluorochrome used (e.g. CD4-FITC).
- [x] The axis scales are clearly visible. Include numbers along axes only for bottom left plot of group (a 'group' is an analysis of identical markers).
- [x] All plots are contour plots with outliers or pseudocolor plots.
- [x] A numerical value for number of cells or percentage (with statistics) is provided.

Methodology

Sample preparation	Populations of cardiomyocytes were harvested and pelleted by centrifugation at 300 xg for 3 mins. Pellets were resuspended in PBS supplemented with 0.1 % BSA and 2 mM EDTA (PBE), with CD90 (Thy-1) Monoclonal Antibody directly conjugated to PE diluted at 1:50, for 1 hour at 4 °C. Cells were then washed with PBE and resuspended in Fixation/Solubilization solution (BD Cytofix/Cytoperm Fixation/Permeabilization Kit, Biosciences) for 20 mins at 4 °C. Following incubation, cells were washed using 1X BD Perm/Wash Buffer (Biosciences) and then resuspended in 1X BD Perm/Wash Buffer containing directly conjugated Anti-Cardiac Troponin T-APC antibody diluted at 1:50 and incubated for 2 hours at 4 °C. Cells were then washed in 1X BD Perm/Wash Buffer, resuspended in PBE and transferred to flow tubes.
Instrument	LSRFortessa Cell Analyzer (BD Biosciences)
Software	FlowJo v10.8.1 software
Cell population abundance	80 ± 9 % of wild-type hESC-CM cells stained positive for the cardiac specific marker troponin T, in agreement with previous studies (Macrae et al., 2021 https://doi.org/10.1093/cvr/cvac065).
Gating strategy	Information on antisera used is provided in the Methods and Reporting Summary. The sample preparation is given in the Methods. A figure exemplifying the gating strategy is provided in the Supplementary information, together with a detailed explanation of the figure in the legend. .

☒ Tick this box to confirm that a figure exemplifying the gating strategy is provided in the Supplementary Information.
